# Low level of MAp44, an inhibitor of the lectin complement pathway, and long-term graft and patient survival; a cohort study of 382 kidney recipients

**DOI:** 10.1186/s12882-016-0373-9

**Published:** 2016-10-18

**Authors:** Julia Smedbråten, Geir Mjøen, Anders Hartmann, Anders Åsberg, Halvor Rollag, Tom Eirik Mollnes, Leiv Sandvik, Morten W. Fagerland, Steffen Thiel, Solbjørg Sagedal

**Affiliations:** 1 Department of Nephrology, Ullevål Oslo University Hospital, Postbox 4950, Nydalen, 0424 Oslo, Norway; 2 Faculty of Medicine, University of Oslo, Oslo, Norway; 3 Department of Transplant Medicine, Rikshospitalet Oslo University Hospital, Oslo, Norway; 4 Norwegian Renal Registry, Oslo University Hospital, Oslo, Norway; 5 School of Pharmacy, University of Oslo, Oslo, Norway; 6 Department of Microbiology, Rikshospitalet Oslo University Hospital, Oslo, Norway; 7 Department of Immunology, Rikshospitalet Oslo University Hospital and K.G Jebsen IRC, University of Oslo, Oslo, Norway; 8 Research Laboratory, Nordland Hospital, Bodø, and Faculty of Health Sciences, K.G.Jebsen TREC, University of Tromsø, Tromsø, Norway; 9 Center of Molecular Inflammation Research, Norwegian University of Science and Technology, Trondheim, Norway; 10 Oslo Centre for Biostatistics and Epidemiology, Research Support Services, Oslo University Hospital, Oslo, Norway; 11 Department of Biomedicine, Aarhus University, Aarhus, Denmark

**Keywords:** Kidney transplantation, Complement, Recipient survival

## Abstract

**Background:**

Higher incidence of malignancy and infectious diseases in kidney transplant recipients is related to immunosuppressive treatment after transplantation and the recipient’s native immune system. The complement system is an essential component of the innate immunity. The aim of the present study was to investigate the association of effector molecules of the lectin complement pathway with graft and patient survival after kidney transplantation.

**Methods:**

Two mannan-binding lectin (MBL) associated proteases, MASP-2 and MASP-3 (activators of the lectin pathway) and two MBL-associated proteins, MAp44 and MAp19 (inhibitors of the lectin pathway) were measured at the time of transplantation in 382 patients (≥17 years old) transplanted in 2000–2001. The cohort was followed until December 31, 2014. Data on patient and graft survival were obtained from the Norwegian Renal Registry. Cox proportional hazard regression models were performed for survival analyses.

**Results:**

Low MAp44 level (1st versus 2–4 quartile) was significantly associated with overall mortality; HR 1.52, 95 % CI 1.08–2.14, *p* = 0.017. In the sub analyses in groups below and above median age (51.7 years), low MAp44 as a predictor of overall mortality was statistically significant only in recipients of ≤51.7 years; HR 2.57, 95 % CI 1.42–4.66, *p* = 0.002. Furthermore, low MAp44 was associated with mortality due to infectious diseases; HR 2.22, 95 % CI 1.11–4.41, *p* = 0.023. There was no association between MASP-2, MASP-3 or MAp19 levels and patient mortality. No association between any measured biomarkers and death censored graft loss was found.

**Conclusions:**

Low MAp44 level at the time of transplantation was associated with increased overall mortality in kidney recipients of median age of 51.7 years or below and with mortality due to infectious diseases in the whole patient cohort after nearly 14-years of follow up after transplantation. No associations between other effector molecules; MASP-2, MASP-3 or MAp19 and recipient mortality were found, as well as no association of any biomarker with death censored graft loss.

**Electronic supplementary material:**

The online version of this article (doi:10.1186/s12882-016-0373-9) contains supplementary material, which is available to authorized users.

## Background

Kidney transplantation is the treatment of choice for end-stage kidney disease, providing patients with a better quality of life and reducing overall morbidity. Despite this, kidney transplant recipients have higher mortality compared to the general population. The effectiveness of the adaptive immune system begins to diminish already with progression of chronic kidney disease. An observed increased incidence of malignancy [1] and infectious diseases [2] after transplantation could be related both to immunosuppressive treatment after transplantation and to the state of the recipient’s immune system.

The complement system is an essential component of the innate immune system. It plays an important role in anti-microbial defense processes such as immunological response to pathogens, but it is also involved in inflammatory processes such as ischemia reperfusion injury, transplant immunity, auto-immune diseases, coagulation [3–5] and probably in development of diabetic angiopathy [6]. The complement system can be activated through the classical, the lectin and the alternative pathways, which are described in details elsewhere [7]. The lectin pathway of the complement system is activated when pattern recognition molecules (PRMs), including the two collectins (mannan-binding lectin (MBL) and collectin-LK (CL-LK)) and the three ficolins (ficolin-1, ficolin-2 and ficolin-3) bind to a fitting pattern on microorganisms or on altered tissues. When this occurs three MBL-associated proteases (MASP-1, MASP-2 and MASP-3) provide activation of the complement system and two MBL-associated proteins (MAp44 and MAp19) serve as natural endogenous competitive inhibitors [7, 8]. MASP-1, MASP-3 and MAp44 arise from the *MASP1* gene by mutually exclusive splicing [9]. In a similar manner MASP-2 and MAp19 arise from the *MASP2* gene [7].

Our hypothesis is that the activity of the native immune system plays an important role in the transplant population given the reduced activity in the adaptive immune system due to immunosuppressive treatment. The aim of this study was to investigate whether the status of the lectin pathway at the time of transplantation may influence long term kidney graft and patient survival. The lectin pathway was investigated by measuring levels of activators like MASP-2 and MASP-3 and of the regulatory molecules MAp44 and MAp19, and by evaluating a possible correlation between levels of MASP-3 and MAp44 or between MASP-2 and MAp19.

## Methods

### Study population

A cohort of 402 adult kidney graft recipients (≥17 years), transplanted in 2000 and 2001 at Oslo University Hospital Rikshospitalet, was included in the original study previously described in detail [10]. Blood samples at the time of transplantation were available in 382 of the patients, who were included in the present study. In the present study the follow up period was extended until December 31, 2014. Data on patient survival was obtained from the Norwegian Renal Registry. Prophylaxis with trimethoprim-sulfamethoxazole against *Pneumocystis jiroveci* was routinely used for 6 months after transplantation. No patients received prophylaxis against cytomegalovirus (CMV) but were treated with valganciclovir at first positive CMV antigen test.

### Immunosuppressive treatment

The immunosuppressive regimen was routinely based on a calcineurin inhibitor, except for five patients with haemolytic uraemic syndrome who received sirolimus. At that time the induction therapy was not included in the standard immunosuppression protocol. Calcineurin inhibitors were combined with either mycophenolate mofetil (MMF) or with induction therapy. Altogether 161 patients (42 %) received induction with basiliximab (Simulect®), and in one patient anti-thymocyte globulin was used as induction therapy. The remaining 220 patients (58 %) received MMF. Only 13 patients received quadruple immunosuppression with basiliximab, calcineurin inhibitors, MMF and steroids. Azathioprine in combination with cyclosporine and steroids was given only to three patients. Except for ten patients who participated in the ATLAS trial and followed a steroid free protocol [11], all patients received steroids.

### Biochemical assays

*MASP*-*2*. The MASP-2 assay was previously described in detail [12]. Microtiter wells were coated with 0.5 μg anti-MASP-2 antibody (MAb clone 8B5) in 100 μl PBS overnight at 4 °C. The wells were blocked and washed with buffer. The samples were diluted 75-fold in 1 M NaCl, 10 mM Tris–HCl, 10 mM EDTA, 15 mM NaN_3_, 0.05 % (*v/v*) Tween 20, pH 7.4, containing 0.01 % (*w/v*) heat aggregated human IgG (Beriglobin, incubated 30 min at 63 °C and centrifuged 10 min at 3000 g to remove large aggregates). The heat aggregated human IgG is included to inhibit the influence of possible rheumatoid factors in sandwich type immuno assays. A pool of plasma was used as a standard and three different plasma samples were used as internal controls and included on each microtiter plate used. Following incubation overnight at 4 °C, the wells were washed and incubated for 1.5 h at room temperature with 0.1 μg biotinylated anti-MASP-2 antibody (MAb 6G12), in 100 μl of TBS/Tw/CaCl_2_ (10 mM Tris–HCl, 145 mM NaCl, 5 mM CaCl_2_, 15 mM NaN_3_, 0.05 % (*v/v*) Tween 20, pH 7.4) containing 0.01 % (*w/v*) heat aggregated human IgG and 1 % (*v/v*) bovine serum. The wells were washed followed by incubation with 10 ng europium-labelled streptavidin (Perkin Elmer) in 100 μl of TBS/Tw, 25 μM EDTA for 1 h at room temperature. After wash bound europium was detected by time-resolved fluorometry after the addition of an enhancement solution (Perkin Elmer).

*MASP-3*. The MASP-3 assay we used was described in details by Degn et al. [9]. Microtiter wells were coated with 0.2 μg antibody reacting with MASP-3 (MAb 5F5) in 100 μl PBS overnight at 4 °C. Residual bindings sites in the wells were blocked with HSA (1 mg/ml TBS), and the wells next received the samples diluted 100-fold in Binding buffer (20 mM Tris–HCl, 1 M NaCl, 5 mM CaCl_2_, 1 mg HSA/ml, 15 mM NaN_3_, 0.05 % (*v/v*) Triton X-100) containing 0.01 % (*w/v*) heat aggregated human IgG. A standard curve was made from a pool of citrate plasma from donor blood. The plasma pool was diluted 1/10 followed by serial 3-fold dilutions in Binding buffer. For quality control, each microtiter plate in addition received the same three citrate plasma samples diluted 100-fold. All samples, standards and controls, were tested in duplicate. Following incubation overnight at 4 °C, the wells were washed thrice with TBS/Tw/CaCl_2_ and incubated for 2 h at room temperature with 25 ng biotinylated anti-MASP-3 antibody (MAb 38.12.3) in 100 μl of TBS/Tw/CaCl_2_ containing 1 % (*v/v*) bovine serum. The wells were subsequently washed thrice and then incubated for 1 h with europium-labeled streptavidin (Perkin Elmer) diluted 1000-fold in TBS/Tw, 25 μM EDTA. After washing with TBS/Tw/CaCl_2_ and the addition of enhancement buffer the wells were read by time-resolved fluorometry.

*MAp44*. Levels of MAp44 were determined as previously described [13]. Wells of microtiter plates were coated with 0.5 μg mouse anti-human MAp44 antibody (MAb 2D5) in 100 μl PBS. Wells were subsequently blocked with TBS/Tw. Serum samples, the standard and three quality controls were diluted 40-fold in Binding buffer containing 100 μg/ml of each of heat-aggregated humane IgG, bovine IgG, rat IgG and mouse IgG at pH 7.4. To construct a standard curve, standard citrate plasma samples with known MAp44 concentrations were diluted 1/10 and then a further 7 times two-fold. Samples of 100 μl were added to wells and incubated overnight at 4 °C. After incubation and washing, the wells were incubated with a biotinylated antibody reacting with MAp44 (MAb 4H2). After washing, 1000-fold diluted europium-labeled streptavidin was added; then, after incubation and washing, enhancement buffer was added. The released europium was measured by time-resolved fluorometry. Inter-assay reproducibility was assessed by determining MAp44 in three different control citrate plasma samples.

*MAp19*. The assay for MAp19 was previously described in detail [14]. Wells were coated with 4 μg anti-MAp19 antibody (MAb 6G12) per ml sodium acetate buffer (50 mM Na-acetate, 145 mM NaCl, pH 4.5) o.n. at 4 °C. The wells were then blocked with HSA, 1 mg/ml TBS, washed thrice with TBS/Tw, and incubated o.n. at m temperature with serum samples diluted 20-fold in MAp19 buffer (10 mM Tris–HCl, 1 M NaCl, 10 mM EDTA, 0.05 % Tween 20) containing 100 μg heat-aggregated hIgG per ml, and 100 μg normal rat IgG per ml. After washing the wells, biotinylated antibody reacting with MAp19 (MAb 4D12) was added at 1 μg per ml TBS/Tw containing 1 mg HSA/ml. Following another wash, the wells were developed with europium-streptavidin as described above. A standard curve was prepared by applying a 2-fold serial dilution of a standard serum (8 dilutions) and a buffer control. Along with three internal control sera (high, medium, low), this was included on every plate. All samples, standards and controls were in duplicates.

### Statistical analyses

The activating and inhibitory molecules of the lectin pathway (MASP-2, MASP-3, MAp44 and MAp19) were analyzed as potential predictors for mortality. Because of the unknown pattern of the association between the effector molecules and mortality, we divided the effector molecules into quartiles, and Kaplan-Meier survival plots were made for each variable. Based on the survival plots for MAp44, a cut point was made at the 25 percentile for MAp44, and the variable was dichotomized. Consequently, we studied the effect of Low MAp44 (≤1716 ng/mL; 1st quartile) versus High MAp44 (>1716 ng/mL; 2nd–4th quartile) on kidney graft and patient survival by Cox proportional hazard regression models. A number of relevant variables known to be associated with survival were tested as predictors and potential confounders in univariable COX models: CMV infection during the first 100 days after transplantation (as time-dependent variable), induction therapy with basiliximab, recipient age per year, recipient gender, living donor, donor age per year, preemptive transplantation (previously not taken in renal replacement therapy), coronary heart disease, hypertensive nephropathy and diabetic nephropathy at the time of transplantation.

Explanatory variables with *p* < 0.2 in the univariable analyses were included in a multivariable Cox model for overall mortality. Similar Cox model for overall mortality was conducted for sub cohort of patients that underwent dialysis before transplantation, with dialysis vintage as one of the co-variables. In the multivariable Cox models for cardiovascular mortality and mortality due to infectious diseases in total cohort and in multivariable Cox model for overall mortality in the subsample (median age of 51.7 years or below), fewer explanatory variables were included to avoid overfitting, i.e. spurious effects due to too few events per explanatory variable. Those variables were chosen based on lowest *p*-value in univariable analyses.

For each Cox model, the proportional hazard assumption was checked with a test based on Schoenfeld residuals and found to be adequately met. We tested potential interactions between age and biomarkers in COX analyses. First, univariable COX analyses were performed in groups according to recipient’s age quartiles. Based on these results the cohort was stratified by median age, and subsequent analyses were done separately for patients below and above median age.

Pearson bivariate correlation coefficients were estimated to explore the relationship between effector molecules that arise from same gene by mutually exclusive splicing, e.g. between MASP-3 and MAp44 or between MASP-2 and MAp19. The Pearson chi-squared test was used to compare categorical variables, and median regression was used to compare medians.

The statistical softwares SPSS (SPSS 21) and Stata 14 (StataCorp LP, College Station, TX) were used to perform the statistical analyses.

## Results

*General outcomes*: The cohort was followed for a median observation time of 13.3 (range 0.1–15) years. During this period 157 of 382 (41 %) patients died. Median time to death was 6.8 (range 0.1–14) years. Causes of death were cardiovascular events in 68 patients, infections in 34 patients, malignancy in 28 patients, and 27 patients died of other causes. Baseline characteristics at the time of transplantation for whole cohort are presented in Table 1. No differences across the groups in the frequencies of CMV infection during the first 100 days after transplantation were found: 54 cases (57 %) in the low MAp44 group, and 172 cases (60 %) in the high MAp44 group, *p* = 0.55.

**Table 1:** Baseline characteristics

Variables	Total *n* = 382	Low Map44 (<1717 ng/ml) *n* = 95	High MAp44 (≥1717 ng/ml) *n* = 287	*p* values
Recipient gender, female^a^	139 (36)	28 (30)	111 (39)	0.11
Living Donor^a^	143 (37)	35 (37)	108 (38)	0.89
Preemptive transplantation^a^	70 (18)	22 (23)	48 (17)	0.16
Diabetic nephropathy^a^	55 (14)	17 (18)	38 (13)	0.26
Induction, basiliximab^a^	161 (42)	40 (42)	121 (42)	0.99
Coronary disease^a^	72 (19)	22 (23)	50 (17)	0.22
Hypertensive nephrosclerosis^a^	75 (20)	16 (17)	59 (21)	0.43
Recipient age, years^b^	51.5 (17–80)	54 (18–79)	51 (17–80)	0.54
Donor age, years^b^	45.5 (1–82)	44 (10–75)	46 (1–82)	0.65

^a^Results presented as number of patients (%)

^b^Results presented as median (range)

### Overall mortality

**Fig. 1:**
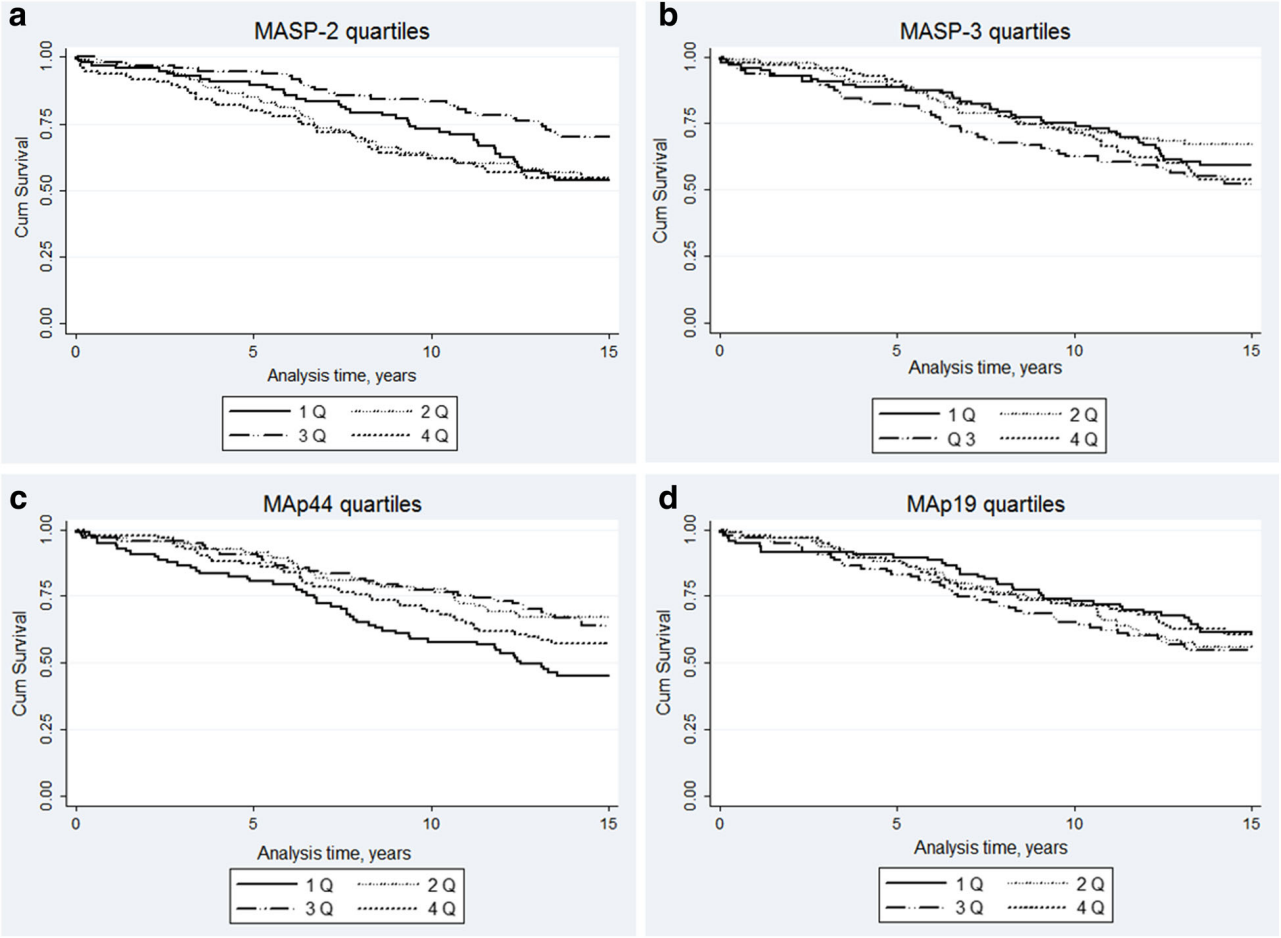
Kaplan-Meier plots for overall mortality. **a** MASP-2 quartiles. Log rank = 0.04. **b** MASP-3 quartiles. Log rank = 0.28. **c** MAP44 quartiles. Log rank = 0.005. **d** MAP19 quartiles. Log Rank = 0.58

*Univariable analyses*: Kaplan-Meier graphs for each tested biomarker are presented in Fig. 1a, b, c and d. Only for MAp44 the survival curve in quartiles diverged significantly, Log rank 0.005.

*MAp44 levels*: Median (IQR) 2057 ng/mL (1716–2398). Cut off value 1716 ng/mL for low versus high MAp44.

**Fig. 2:**
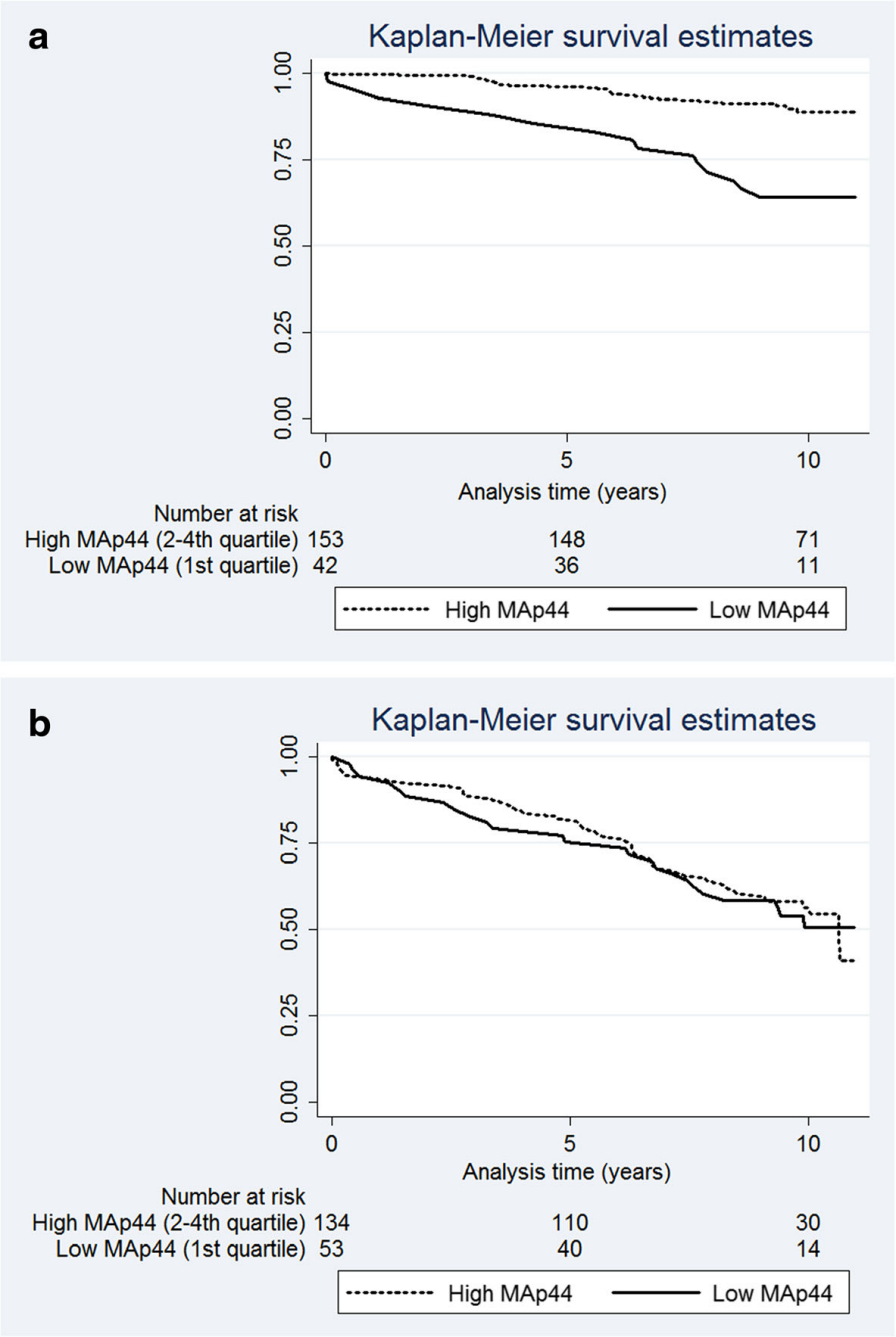
Kaplan-Meier plots for overall mortality in age groups for Low MAp44 versus high MAp44. **a** Patient age ≤51.7 years. **b** Patient age >51.7 years

*Multivariable Cox analyses*: Low MAp44 (≤1716 ng/mL) was significantly associated with mortality in multivariable Cox models with HR 1.52, 95 % CI 1.08–2.14, *p* = 0.017. Other significant risk factors associated with increased mortality were recipient age (per year) and diabetic nephropathy (Table 2). We observed an interaction between MAp44 and age. The patients of median age (51.7 years at the time of transplantation) or below had a statistically significant association of low MAp44 with mortality, while no such association was found for patients above median age (Fig. 2a and b, respectively). There were 195 patients below median age, of which 46 (24 %) died. Among the 187 patients above median age, 111 (59 %) died. In multivariable Cox model for subsample of patients below median age, low MAp44 was significantly associated with mortality, HR 2.57, 95 % CI 1.42–4.66, *p* = 0.002, after adjustment for diabetic nephropathy, coronary heart disease, recipient and donor age (per year) (Table 3). There was no significant difference in mean MAp44 levels in patients ≤51.7 years as compared to those >51.7 years at transplantation. To further examine age dependence we found no difference in mean MAp44 levels in quartiles based on age in a control group of 350 blood donors previously described [9].

**Table 2:** Overall mortality in the total study cohort

Variable	Univariable analyses			Multivariable analysis		
	HR	95 % CI	*p*	HR	95 % CI	*p*
^a^Low MAp44	1.73	1.24–2.41	0.001	1.52	1.08–2.14	0.017
^a^CMV infection	1.46	1.05–2.03	0.025	1.17	0.83–1.66	0.38
Recipient age, per year	1.07	1.05–1.08	<0.001	1.06	1.05–1.08	<0.001
Donor age, per year	1.02	1.01–1.03	0.001	1.01	1.00–1.02	0.08
^a^Living donor	0.57	0.40–0.81	0.002	1.06	0.73–1.54	0.76
^a^Diabetic nephropathy	1.38	0.91–2.09	0.13	2.13	1.39–3.42	<0.001
^a^Coronary heart disease	2.58	1.84–3.62	<0.001	1.37	0.96–1.95	0.85
^a^Hypertensiv nephrosclerosis	1.90	1.34–2.69	<0.001	1.11	0.77–1-60	0.59
^a^Recipient gender, female	1.15	0.83–1.58	0.40			
^a^Preemptive transplantation	0.85	0.56–1.29	0.44			
^a^Induction, basiliximab	1.20	0.88–1.65	0.25			

Results of univariable and multivariable Cox regression models

Low MAp44 1st versus 2–4 quartiles

^a^yes versus no

**Table 3:** Overall mortality in the subsample of patients of median age (51.7 years) or below

Variable	Univariable analyses			Multivariable analysis		
	HR	95 % CI	*p*	HR	95 % CI	*p*
^a^Low MAp44	2.91	1.61–5.27	0.001	2.57	1.42–4.66	0.002
Recipient age, per year	1.05	1.01–1.09	0.012	1.04	1.00–1.08	0.05
Donor age, per year	1.02	0.99–1.04	0.11	1.02	1.00–1.04	0.09
^a^Diabetic nephropathy	2.16	1.14–4.11	0.019	1.93	0.93–3.98	0.08
^a^Coronary heart disease	3.56	1.61–6.57	0.001	1.91	0.88–4.18	0.10
^a^Hypertensiv nephrosclerosis	0.73	0.23–2.34	0.59			
^a^CMV infection	2.42	0.33–17.73	0.38			
^a^Living donor	0.90	0.51–1.61	0.90			
^a^Recipient gender, female	1.45	0.81–2.60	0.21			
^a^Preemptive transplantation	1.08	0.52–2.23	0.84			
^a^Induction, basiliximab	1.04	0.57–1.87	0.91			

Results of univariable and multivariable Cox regression models

Low MAp44 1st versus 2–4 quartiles

^a^yes versus no

Furthermore, the association of MAp44 with overall mortality in a sub cohort of patients that underwent dialysis before transplantation (*n* = 303) was investigated. Median (IQR) dialysis vintage in this sub cohort was 12 (6–21) months and 129 (43 %) of patients died during the follow up period. MAp44 remained significantly associated with mortality, HR 1.72, 95 % CI 1.18–2.51, *p* = 0.005, adjusted for other significant risk factors (Table 4).

**Table 4:** Overall mortality in sub cohort of patients who underwent dialysis before transplantation

Variable	Univariable analyses			Multivariable analysis		
	HR	95 % CI	*p*	HR	95 % CI	*p*
^a^Low MAp44	1.81	1.25–2.61	0.002	1.72	1.18–2.51	0.005
Dialysis vintage, per month	1.02	1.01–1.04	0.002	1.02	1.00–1.03	0.031
Recipient age, per year	1.07	1.05–1.08	<0.001	1.06	1.05–1.08	<0.001
^a^Coronary heart disease	2.73	1.89–3.93	<0.001	1.37	0.92–2.04	0.12
^a^Hypertensive nephropathy	1.72	1.18–2.50	0.04	0.99	0.67–1.50	0.99
Donor age, per year	1.02	1.01–1.03	<0.001	1.01	1.00–1.02	0.074
^a^Living donor	0.56	0.37–0.83	0.004	1.24	0.79–1.95	0.36
^a^Diabetic nephropathy	1.31	0.79–2.18	0.30	2.28	1.31–3.95	0.003

Results of univariable and multivariable Cox regression models

Low MAp44 1st versus 2–4 quartiles

^a^yes versus no

No associations of other effector molecules, MASP-2, MASP-3 or MAp19 with overall mortality were found.

### Mortality due to infectious diseases

Low MAp44 (≤1716 ng/mL) was significantly associated with mortality due to infectious diseases in multivariable Cox analysis, HR 2.22, 95 % CI 1.11–4.41, *p* = 0.023, when adjusted for recipient age (per year), hypertensive nephropathy and coronary heart disease at the time of transplantation (Table 5). We found no interaction between low MAp44 and age in the analysis of mortality due to infectious diseases.

**Table 5:** Mortality due to infections in the total study cohort

Variable	Univariable analyses			Multivariable analysis		
	HR	95 % CI	*p*	HR	95 % CI	*p*
^a^Low MAp44	2.68	1.36–5.28	0.004	2.22	1.11–4.41	0.023
Recipient age, per year	1.08	1.05–1.11	<0.001	1.07	1.04–1.11	<0.001
^a^Coronary heart disease	2.59	1.26–5.32	0.01	1.38	0.65–2.92	0.40
^a^Hypertensive nephropathy	1.91	0.91–4.01	0.09	1.02	0.47–2.21	0.96
Donor age, per year	1.01	0.99–1.03	0.46			
^a^Living donor	0.54	0.52–1.16	0.11			
^a^CMV infection	1.05	0.53–2.08	0.90			
^a^Diabetic nephropathy	0.63	0.19–2.06	0.45			

Results of univariable and multivariable Cox regression models

Low MAp44 1st versus 2–4 quartiles

^a^yes versus no

### Cardiovascular mortality

No association between low MAp44 with increased cardiovascular mortality was found.

### Death censored graft loss

None of effector molecules, MASP-2, MASP-3, MAp44 or MAp19 was associated with increased graft loss.

### Correlation between MASP-3 and MAp44 or MASP-2 and MAp19

We tested if MASP-3 and MAp44 or MASP-2 and MAp19 were correlated. None of these variables was highly correlated. The only significant correlation was between MASP-3 and MAp44 (r = 0.24, *n* = 382, *p* < 0.001); however, the size of the correlation was small. No correlation between MASP-2 and MAp19 was found.

## Discussion

In the present study the associations of several complement biomarkers with long-term kidney graft and recipient survival were investigated. Low MAp44 levels at the time of transplantation were found to be associated with increased overall mortality. The association of low MAp44 with increased mortality was statistically significant only in kidney transplant recipients of median age or younger. It is largely unknown whether MAp44 level alters with age or if its regulatory role is simply more important in younger age. We found no variation in mean MAp44 levels with increasing age neither in the study cohort nor in a control group of healthy blood donors. However, blood samples from adolescents younger than 17 years old were not available in the present study. Further, the median levels of MAp44 in patients in dialysis versus those not receiving dialysis before transplantation were not significantly different and they were comparable with median (IQR) levels of MAp44 in healthy controls in another study, 1938 ng/mL (1250–3836), (Trolborg et al. [15]). Analysis of overall mortality in the sub cohort of patients that underwent dialysis before transplantation showed a significant association of low MAp44 with mortality when adjusted for dialysis vintage in months and other relevant co-variables. Low MAp44 was in addition strongly associated with mortality due to infectious disease. This finding is very interesting since in principle the proteins of the lectin pathway participate in the microbial defense. Low level of MAp44, the inhibitor of the lectin pathway, is supposed to lead to increased activity in the lectin complement pathway and hence better immunological response on pathogens. Still, the results may indicate that the excessive activation of the complement system has potentially detrimental effects. The level of mRNA encoding MAp44 in human tissues has the highest relative expression in the heart, followed by much weaker expression in liver and brain [13]. High expression of MAp44 in heart musculature has therefore been supposed to prevent complement-induced heart damage [16]. However, it may not be the only mechanism according to the finding in the present study, since low MAp44 was associated with overall mortality and mortality due to infectious disease, but not with cardiovascular mortality. Obviously, both the regulatory mechanism of MAp44 and potential longitudinal changes in MAp44 levels during progression of chronic kidney failure and after transplantation remain to be thoroughly investigated.

MASP- 2 is a key molecule that binds to MBL and ficolins and provides activation of the lectin pathway [7]. Several previous studies have shown that excessive lectin pathway activation may have an adverse impact and inhibition of MASP-2 may have a beneficial effect. Murine model studies by Schwaeble et al. and Asgari et al. demonstrated a protective role of MASP-2 deficiency in myocardial, gastrointestinal and renal ischemia reperfusion injury [17, 18]. In addition, the injection of the murine-specific MASP-2 inhibitor significantly reduced size of tissue damages [18]. In a reasonably large study of 605 patients with colorectal cancer and 150 healthy blood donors as controls, Ytting et al. found a significant association between high preoperative MASP-2 levels and increased risk of both cancer recurrence and mortality [19]. However, we found no association of pre-transplant levels of other effector molecules; MASP-2, MASP-3 or MAp19 with long-term survival after kidney transplantation.

The mutations in the *MASP1* gene, encoding the three splice products MASP-1, MASP-3 and MAp44, were linked to autosomal-recessive syndrome 3MC, characterized by growth and mental retardation, characteristic facial dysmorphism and skeletal anomalies [4, 20]. Since MASP-1, MASP-3 and MAp44 arise from *MASP1* gene by mutually exclusive splicing [7], we investigated whether there was any correlation between levels of these splice products. In the present study a weak but statistically significant correlation between the MAp44 and MASP-3 levels was found. A previous study of 200 adult Danish blood donors did not reveal such a correlation [9]. In the same manner MASP-2 and MAp19 arise from the *MASP2* gene by mutually exclusive splicing. We found no correlation between levels of these two proteins in our cohort.

The strength of the present study is a near 14 years follow up of the complete cohort of kidney transplant recipient, where no patients were lost in follow-up. The reasonable size of the study cohort gives adequate statistical power. However, it is an observational study and as such shows association but not causality. It remains to be elucidated if the effector molecules play a pathogenic role or are simply markers of adverse outcomes. The present study doesn’t take into account possible changes in MAp44 levels that may take place after transplantation, and this also may represent a limitation of the study. The changes in the biomarker levels after transplantation should be a topic of investigation in future studies.

## Conclusions

Low MAp44 level at the time of transplantation was associated with increased overall mortality and mortality due to infectious diseases in kidney recipients after nearly 14-years of follow up after transplantation. The adverse impact of low MAp44 was only statistically significant in younger kidney recipients, in median age of 51.7 years or below. No associations between other effector molecules; MASP-2, MASP-3 or MAp19 and recipient mortality were found, as well as no association of any biomarker with death censored graft loss. These findings provide new information about the role of the lectin complement pathway in kidney transplanted recipients, a field that is poorly explored and contains the information mostly collected from animal models. However, the findings of the present study should be interpreted with caution and studies of the possible mechanisms of adverse effects on long-term survival are needed.

## Additional file

Additional file 1: Biomarkers levels in quartiles. (XLSX 21 kb)

## Abbreviations

CL-LK: Collectin-LK
CMV: Cytomegalovirus
MAp19: Mannan-binding lectin associated protein 19
MAp44: Mannan-binding lectin associated protein 44
MASP-2: Mannan-binding lectin associated protease-2
MASP-3: Mannan-binding lectin associated protease-3
MBL: Mannan-binding lectin
MMF: Mycophenolate mofetil
PRMs: Pattern recognition molecules
